# Volatile Gas Sensing through Terahertz Pipe Waveguide

**DOI:** 10.3390/s20216268

**Published:** 2020-11-03

**Authors:** Ja-Yu Lu, Borwen You, Jiun-You Wang, Sheng-Syong Jhuo, Tun-Yao Hung, Chin-Ping Yu

**Affiliations:** 1 Department of Photonics, National Cheng Kung University, No. 1 University Road, Tainan 70101, Taiwan; l76071257@ncku.edu.tw (J.-Y.W.); l76094108@gs.ncku.edu.tw (S.-S.J.); 2 Department of Applied Physics, Faculty of Pure and Applied Sciences, University of Tsukuba, 1-1-1 Tennodai, Tsukuba, Ibaraki 305-8577, Japan; 3 Department of Photonics, National Sun Yat-sen University, Kaohsiung 80424, Taiwan; b053090020@student.nsysu.edu.tw (T.-Y.H.); cpyu@faculty.nsysu.edu.tw (C.-P.Y.)

**Keywords:** submillimeter wave, terahertz wave, anti-resonant reflection optical waveguide (ARROW), waveguide sensor, optical gas sensing

## Abstract

Gas sensing to recognize volatile liquids is successfully conducted through pipe-guided terahertz (THz) radiation in a reflective and label-free manner. The hollow core of a pipe waveguide can efficiently deliver the sensing probe of the THz confined waveguide fields to any place where dangerous vapors exist. Target vapors that naturally diffuse from a sample site into the pipe core can be detected based on strong interaction between the probe and analyte. The power variation of the THz reflectance spectrum in response to various types and densities of vapors are characterized experimentally using a glass pipe. The most sensitive THz frequency of the pipe waveguide can recognize vapors with a resolution at a low part-per-million level. The investigation found that the sensitivity of the pipe-waveguide sensing scheme is dependent on the vapor absorption strength, which is strongly related to the molecular amount and properties including the dipole moment and mass of a gas molecule.

## 1. Introduction

Remote sensing of gas is important to address security concerns in environmental monitoring, industrial gas-leak inspection, and breath detection. Dielectric pipes are popularly used to transport or collect gas analytes [1]. Assembling gas-sensing units with a fluidic pipe is straightforwardly useful for in situ and remote detection. Among studies on gas sensing, the detections of the gaseous specimen are based mainly on the electrical and chemical properties or the absorption spectrum using an optical method in the infrared (IR) regime. Electrochemical gas sensors require specific substances to adsorb and trace the gas analytes [2,3,4,5,6]. These sensors have some drawbacks such as poor labelling uniformity, low stability, long-term reaction, and heat treatment. As a result, electrochemical gas sensors have difficulty maintaining high detection accuracy in harsh surroundings.

Gas sensing based on optical methods is relatively simple without adsorption media or molecular labeling [7]. As the vibrational transitions of gaseous specimens are usually located in the near- or mid-IR band, the advantages of optical gas sensors are highly selective based on the molecular signature spectra. Particularly for fiber-waveguide schemes, the gas, which fills in the hollow core of a fiber waveguide and meets the guided wave, can be identified by analyzing the variations of transmittance or reflectance of the guided wave [8,9]. A low concentration of gas, equal to or less than several parts in 10^6^ (parts per million, ppm), can thus be identified.

These minute amounts of analytes in waveguide sensing are operated based on the largest field–analyte overlapping volumes and matched photon-energy of radiation at certain spectral frequencies. For example, the IR wave is guided by the hollow-core fiber (HCF) waveguide to excite the intramolecular vibrations of gas, and the transmission power at the characteristic frequency strongly attenuates through the energy-level transition (i.e., fingerprint absorption spectrum [10]). At the IR regime, an external pump or high-pressure tank is needed to inject the gas into an IR HCF waveguide because of the small core size. The fiber length is as long as several meters to achieve the ppm-level sensitivity [11,12]. The fiber-coupled substrate-integrated hollow waveguides overcome the restriction of gas diffusion using sufficiently large waveguide core sizes [13] and their detection limits are higher than several tens of ppm. Although the gas samples can naturally diffuse without external pump instruments to reduce the response times of gas sensing, the sensing capability obviously degrades. This results from the short light–analyte interaction length of around 5–13 cm, given that the gas chamber should be engineered on a chip substrate [13].

To detect the optical absorption of gas that results from low-frequency vibration or rotation, the HCF-guided radiation frequency should be as low as 0.1–10 THz, called THz radiation (or THz waves) [14,15]. For example, the THz pipe waveguides, used as one of the low-loss HCF waveguides in the THz frequency regime, have relatively large core sizes of several millimeters and are experimentally demonstrated from various dielectric pipes that are widely used in household fluidic systems or disposable breathing circuits [16]. Compared with gas detection using an IR fiber with a core at a micrometer scale [17], gas sensing through THz fibers is advantageous as it easily achieves the largest overlapping volumes in a simple way, and to diffuse gas in the HCF without need for an air-pumping source. Thus, remotely and in situ sensing of gas analytes can be achieved within one large-core fluidic pipe at THz frequency regime to reduce a complicated system into a relatively simple and compact sensing unit. However, one common drawback among THz gas sensing approaches is that the dipole moment associated with the molecular energy transitions is usually weak for molecules with low polarity, which leads to weak absorption of THz radiation. Moreover, a long gas cell is normally required to obtain an observable absorption [18,19]. Two methods can be used for insufficient molecular absorption strength and to maximize the field–analyte interaction volume. One is to design a THz fiber with a moderate modal confinement and a low propagation loss. The other one is to insert a porous structure in a THz spectroscopic system [20].

Volatile organic compounds (VOCs), which are produced from most industrial processes, are harmful to human organs. A growing number of industries are being required to reduce their VOC generation. Most VOCs are flammable and colorless substances. For example, acetone vapor with a volume concentration of approximately 2.5–12.8% mixed in ambient air is easily ignited by fire or any electrostatic discharge [21]. Utilizing the fingerprint absorption in the THz regime to identify hazardous VOCs is safer than electronic and chemical sensing schemes without any electric spark risk. 

Experimentally identifying VOC vapors through free space propagation of THz radiation in a bulky gas cell has been demonstrated in a process, called terahertz gas-phase spectroscopy [18]. A 60 cm-long THz HCF used as a gas cell to guide THz radiation and vapor is presented to distinguish the ammonia and water vapors [19]. However, the transmission sensing schemes presented by the THz gas-phase spectroscopy [18] and a THz HCF [19] cannot be operated in a reflective manner for the remote and in situ detection. A photonic crystal fiber used as one of the THz HCF waveguides for sensing VOCs is theoretically demonstrated, and the minimum detectable concentration is at the ppm level [22]. Most of the gas molecules have molecular absorption beyond 1 THz, which can be detected by specially designed THz resonator devices to monitor power loss [23] or by free-space illumination with broadband THz radiation to measure fingerprint spectra [18,24,25]. At the sub-millimeter wave range of 0.3–0.4 THz, gas molecules have relatively weak THz absorption and are not easily detected from the THz power variation. Developing gas-sensing applications at the communication band of 0.3–0.4 THz is thus difficult. 

In the work, different vapor densities and categories with minute amounts were experimentally identified by 0.3–0.4 THz waves using a glass pipe waveguide in a reflective manner. Reflectivity spectrum of round-trip propagation was first characterized for this pipe-sensing scheme to study the effects of waveguide modes on vapor sensing. Acetone vapor was used as a standard gas sample for studying the frequency-dependent THz signal response and sensitivity calibration. At the most sensitive THz waveguide frequency, the highest sensitivity of vapor concentration change for acetone detection was measured down to 4 ppm with a THz linear response of 36–110 ppm vapor density, which was better than the rate reported in previous studies [19,24,25]. The study proved that the critical factor of a highly sensitive HCF gas sensor not only involves a sufficiently long fiber length, but also the high confinement of the guided radiation inside the core. Thus, the feasibility of remote and in situ sensing gases is potentially workable on the basis of a dielectric pipe and submillimeter waves at the THz communication band.

## 2. Materials and Methods

### 2.1. Optical Configuration

**Figure 1:**
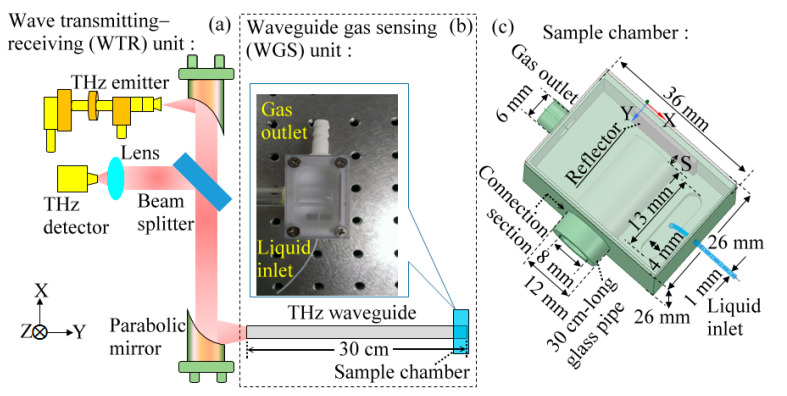
Configurations of (**a**) wave transmitting–receiving (WTR) and (**b**) waveguide-gas-sensing (WGS) units. (**c**) Mechanical assembly of a sample chamber, a glass pipe, a metal reflector, and tubing kits.

A THz gas-sensing system based on a pipe waveguide is illustrated in Figure 1. The system was constructed by a wave transmitting–receiving (WTR) unit and a waveguide-gas-sensing (WGS) unit. Figure 1a schematically plots the components of a WTR unit including an electric THz emitter, an electric THz detector, a lens, two parabolic mirrors, and a beam splitter.

A Gunn oscillator module works as the emitter to radiate THz continuous waves from 0.316 THz to 0.408 THz. The frequency sweeping in the range of 0.316–0.408 THz was realized by two mechanical actuators of the Gunn oscillator module with a swept frequency resolution of 4 GHz. The radiation power range was 0.09–0.21 mW, and the spectral peak was at 0.340 THz with a power signal-to-noise ratio (SNR) of 10^4^. The high SNR was achieved through THz wave detection including a zero-bias Schottky diode, a lock-in amplifier, and a mechanical chopper. The zero-bias Schottky diode was used as a THz wave detector. In front of the detector, THz waves were modulated by a mechanical chopper at a frequency of 220 Hz, which was synchronous to the lock-in amplifier. Thus, the noise was suppressed to perform a high SNR for the THz-wave detection. A pair of off-axis parabolic mirrors was used to collimate and focus the THz waves into a THz waveguide. To receive the reflected THz wave from the waveguide, a high-resistance silicon plate was used as a beam splitter that collects the reflected THz waves into the detector.

The WGS unit, as shown in Figure 1b, involves a THz pipe waveguide and a sample chamber for vapor generation that mimics the distant gas analyte under test. The focused THz waves from a parabolic mirror were coupled into a 30 cm-long THz pipe waveguide. THz waves were transmitted from the pipe waveguide and then entered a sample chamber. A reflector attached inside the sample chamber can straightforwardly reflect the guided waves back into the pipe waveguide. The mechanical assembly between a THz waveguide and a sample chamber in the WGS unit is shown in the inset of Figure 1b, which presents a photograph of the top-view configuration. In the gas-sensing experiment, a liquid inlet and gas outlet were machined on the sample chamber to manipulate volatile liquids and their vapor gases.

Figure 1c illustrates the mechanical construction and dimensions of a THz waveguide, a sample chamber, a liquid inlet, and a gas outlet. The THz waveguide is a hollow-core pipe that is constructed using a 2 mm-thick glass pipe wall and an 8 mm inner-core diameter. The sample chamber reflector is a square aluminum plate with an 18 mm width. There is a free-running distance, denoted as *S* in Figure 1c, between the pipe-output end and chamber reflector. Based on the theory of beam optics, the pipe-output beam along the free-running space has an approximately uniform beam size with a 8 mm beam diameter or a very small divergent angle (*β*) when the *S* value is several millimeters within the beam Rayleigh range of 11.3 mm. That is, the pipe-output beam spot through a free-running distance of several millimeters can be fully covered by the reflector. 

As the connection section between the pipe waveguide and the chamber, indicated in Figure 1c, is not mechanically fixed, the *S* value can be changed for different sensing experiments or rearranging the WGS unit. Such variation of a *S* value could possibly cause a slight angle variation of a partial ray trace back into the pipe. The THz reflectivity or reflected power is therefore changed by rearranging the WGS unit. In contrast, for the reflectivity measurement of the same sensing experiment, the setup parameters of the WGS unit, *S* and *β*, are kept fixed to reduce the power deviation for sample recognition.

The sample chamber was made of Teflon material and had a width of 26 mm, a length of 36 mm, and a height of 26 mm. The widths of the liquid inlet and gas outlet were 1 mm and 6 mm, respectively, fitting the external tube connection. A liquid fluidic channel was machined at the bottom of the sample chamber, which had a length, width, and depth of 13, 4 and 4 mm, respectively. The liquid channel was externally connected with a 1 mm-wide polyethylene (PE) tube through the liquid inlet to receive volatile liquids. An air pump with a flow rate of 23 L/min connected the 6 mm-wide gas outlet to exhaust the vapor or liquid residues for precision detection. During the gas-sensing process, the sample loading, natural volatilization, and sensing processes were performed at room temperature and normal atmosphere without any active layer and external pumping source to adsorb the analytes.

### 2.2. Wave-Guidance Principle of a Dielectric Pipe

The gas analytes in a WGS unit are filled with the hollow-core space of the glass pipe and can be sufficiently covered by the wave guiding field. However, the THz waves that radiate from the WTR unit (Figure 1a) are not all guided along the glass pipe. The glass pipe waveguide used in the experiment was based on the principle of antiresonant reflecting wave guidance [16]. The antiresonant waves are confined inside the hollow core and are thus called core modes; the resonant waves are called cladding modes because their wavelengths exactly match the Fabry–Pérot resonance (FPR) criterion of a pipe-wall cladding. Although the power of cladding waves is almost depleted from FPR, the cladding waves can still be detected in the experiment because partial evanescent fields of FPR leak toward the hollow core to be guided. The frequencies of the core and cladding modes are individually defined in Equations (1) and (2), which are denoted as *ν_cor_* and *ν_cld_*, as follows:

(1) $$\nu_{cor}=C(2m+1)/4d\sqrt{n_{1}^{2}-n_{0}^{2}}$$

(2) $$\nu_{cld}=mC/2d\sqrt{n_{1}^{2}-n_{0}^{2}}$$

where *C*, *m*, *d*, *n*_1_, and *n*_0_ represent light speed in the air, a mode order, thickness of a pipe wall, refractive indices of the pipe wall, and a hollow core (*n*_0_ = 1.0), respectively.

### 2.3. Gas-Sensing Mechanism

The complex amplitude of the glass-pipe-waveguide field is described as

(3) $$\left|\vec{E}\right|=\left|E\left(x,z\right)\right|e^{-jky}$$

where *k* is the propagation constant of a THz wave and defined as follows [26]:

(4) $$k=k_{0}\sqrt{1+\chi }$$

where *k*_0_ and χ are, respectively, a free-space wavenumber and an electric susceptibility at the hollow-core space of the glass pipe. For the analyte-filled hollow core, the *k* parameter of a THz wave is a complex value as shown in the following equation:

(5) $$k=n-j\left(\alpha /k_{0}\right)=\sqrt{1+\chi^{\prime }+j\chi^{\prime \prime }}$$

where n, α, $\chi^{\prime }$ , and $\chi^{\prime \prime }$ are a refractive index, an absorption coefficient, and the real and imaginary parts of an electric susceptibility (χ), respectively. The vapor belongs to a weak absorption material except at the fingerprint spectral frequencies (i.e., spectral absorption lines) [26]. Thus, the corresponding *n* and *α* can be simplified from a Taylor series in Equation (5), as shown in the following equations:

(6) $$n=1+\chi^{\prime }/2$$

(7) $$\alpha =-k_{0}\chi^{\prime \prime }/2$$

The refractive index of a THz wave (*n*) linearly relates to the real part of susceptibility, and the THz absorption coefficient (*α*) is proportional to the imaginary part of susceptibility. Furthermore, the dielectric polarization density of a vapor molecule ( $\vec{P}$ ), responding to the pipe-waveguide field ( $\vec{E}$ ), is presented as follows [26]:

(8) $$\vec{P}=\epsilon_{0}\left(\chi^{\prime }+j\chi^{\prime \prime }\right)\vec{E}\propto \rho \vec{p}$$

where *ρ* and $\vec{p}$ are molecular density (i.e., molecular amounts per unit volume) and an electric dipole moment of the vapor gas, respectively. In Equations (3)–(7), the molecular absorption coefficients (*α*) at non-characteristic frequencies, departing from the fingerprint absorption frequencies, are proportionally increased with the vapor amount (*ρ*) and the molecular dipole moment ( $\vec{p}$ ), which depends on the vapor category. The vapor absorption in the THz frequency regime arises from the rotational level transition that is sensitive to molecular structures and properties such as the electric dipole moment and mass of a molecule.

### 2.4. Gas-Sensing Parameters

The THz waves in the WGS unit are attenuated by gas analytes (Equation (7)) when the WGS reflectance is compared between the sample-loaded and blank conditions. This approach is an intensity interrogation sensing method [11,12]. In the sensing experiment, the reflectance of a blank WGS unit is expressed as

(9) $$R_{0}=P_{air}/P_{0}=\delta e^{{-\alpha }_{0}L}$$

where *P*_0_, *P_air_*, *α*_0_, and *R*_0_ are respectively the input THz wave power, reflected THz wave power, absorption coefficient, and reflectance of the blank WGS unit (i.e., without sample loading in the WGS). *L* represents the round-trip length of a glass pipe waveguide, corresponding to a double waveguide length and equal to 60 cm. *δ* represents the coupling efficiency while THz waves input the glass pipe from the free air space. Similarly, the reflectance for sensing gas analytes inside the WGS unit is defined as

(10) $$R_{\%}=P_{\%}/P_{0}=\delta e^{-\alpha_{\%}L}$$

where *α*_%_, *P*_%_, and *R*_%_ are absorption coefficient, reflected THz wave power, and reflectance of a sample-loaded WGS unit, respectively. The absorption losses of a WGS unit, *α*_0_, and *α*_%_, result from three factors: THz wave absorption of air or gas along the guidance path, loss of the metal reflector, and round-trip waveguide loss of the glass pipe. When the latter two losses from the metal reflector and glass pipe waveguide are sufficiently low, both values can be neglected and the sample and air absorption losses of *α*_%_ and *α*_0_ become dominant. The difference of WGS loss with and without the gas analyte is consequently derived from Equations (9) and (10), as shown in the following:

(11) $$\alpha_{\%}-\alpha_{0}=ln\left(R_{0}/R_{\%}\right)/L$$

For the THz waves, *α*_0_ is in the order of 10^−5^ cm^−1^ [27], which is sufficiently small and can be ignored for sensing a vapor. Thus, the loss coefficient of the vapor can be expressed as follows:

(12) $$\alpha_{\%}=ln\left(R_{0}/R_{\%}\right)/L$$

The values of *R*_0_, *R*_%_, and *α*_%_ are discussed in this presentation for THz-frequency-dependent reflectivity and responsivity, which are associated with the capabilities of waveguide confinement and sensing. Aside from *α*_%_, another sensing parameter, Δ*Re.*, is introduced to recognize various gas analytes via core-mode reflection and characterize the responsivity of pipe-cladding modes. Δ*Re.* denotes the reflectance difference between the blank and sample-loaded WGS units (Δ*Re.* = *R*_%_*–R*_0_), which is mainly due to the THz-wave power attenuation caused by vapor absorption. Compared with *α*_%_, Δ*Re.* is relatively simple for the waveguide-based sensing scheme to distinguish various types and amounts of vapors in a remote and in situ manner.

### 2.5. Preparation of Gas Samples

Four different types of volatile liquids, namely, acetone (*CH_3_COCH_3_*) (Anaqua Chemicals Supply, model no. AE-1011), ammonia (*NH_4_OH*) (Sigma Aldrich, model no. 221228), hydrochloric acid (*HCl*) (Sigma Aldrich, model no. H1758), and methanol (*CH_3_OH*) (Merck KGaA, model no. 113351) were prepared for their vapor gas sensing based on a THz pipe waveguide (Figure 1). The injected volume of volatile liquid was fixed at 0.3 cm^3^ by a syringe, which was connected to the PE tubing inlet (Figure 1c). According to Raoult’s law [28], the vapor pressure of the WGS unit is approximately proportional to the volume concentration of a volatile aqueous solution. Thus, different saturation pressures of vapors are produced for the same liquid volume (0.3 cm^3^). In the same volume of an enclosed WGS unit, different saturation pressures correspond to various amounts of vapor molecules. Therefore, the vapor densities (*ρ*) at different saturation vapor pressures are estimated according to the assumption of an ideal gas equation [20]. In the experiment, the volume concentration ranges of acetone and ammonia aqueous solutions were 0.125–100% and 1.75–28%, respectively. Thus, the corresponding *ρ* ranges of acetone and ammonia vapors were 29–765 and 17–959 ppm, respectively. Based on the molecular property of a large molecular dipole moment [20], the acetone vapor was used as a standard vapor sample to study the frequency-dependent sensing abilities of the WGS unit and calibrate the dynamic response and recovery times under various vapor densities.

## 3. Results and Discussion

### 3.1. Reflectivity Spectrum of Round-Trip Propagation

**Figure 2:**
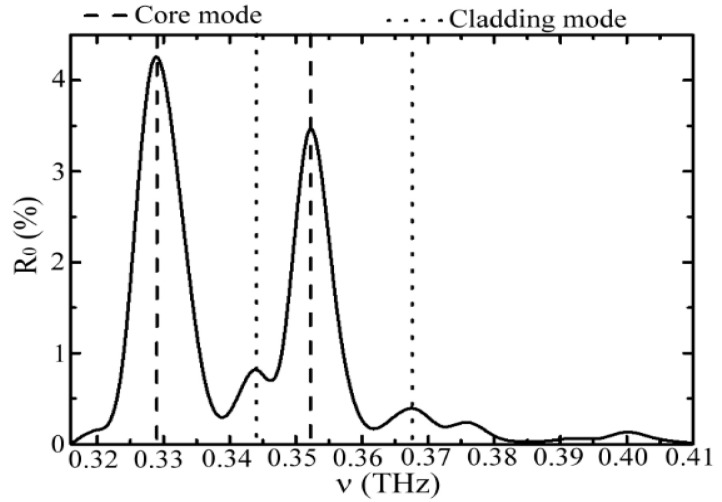
Reflectivity spectrum of a blank waveguide-gas-sensing (WGS) unit in the experiment.

The measured reflectivity (*R*_0_) spectrum of the blank WGS unit is illustrated in Figure 2. The two highest reflectivity peaks are denoted by the dashed lines at the 0.329 THz and 0.352 THz frequencies and correspond to the 10th- and 11th-order core modes of the glass pipe, respectively. These results were estimated from the values of 2 mm-*d*, 2.6-*n*_1_, and 1.0-*n*_0_, based on Equation (1). In addition to the two main peaks, two secondary peaks at 0.344 THz and 0.368 THz frequencies were detected after the round-trip propagation in the pipe core, denoted by the dotted lines in Figure 2. The spectral frequencies of secondary peaks approximate those of the 11th- and 12th-order cladding modes (Equation (2)) with a reflectivity of 0.39–0.84%. The reflectivity was much lower than that of the core mode of 3.48–4.27%. However, less or zero reflected power of the core and cladding modes were found at a high-frequency range of 0.370–0.408 THz (Figure 2). This condition resulted from the weak incident power (less than 0.1 mW) detected by the WTR unit after the wave propagation of a double path length.

### 3.2. Frequency-Dependent Sensing Abilities

**Figure 3:**
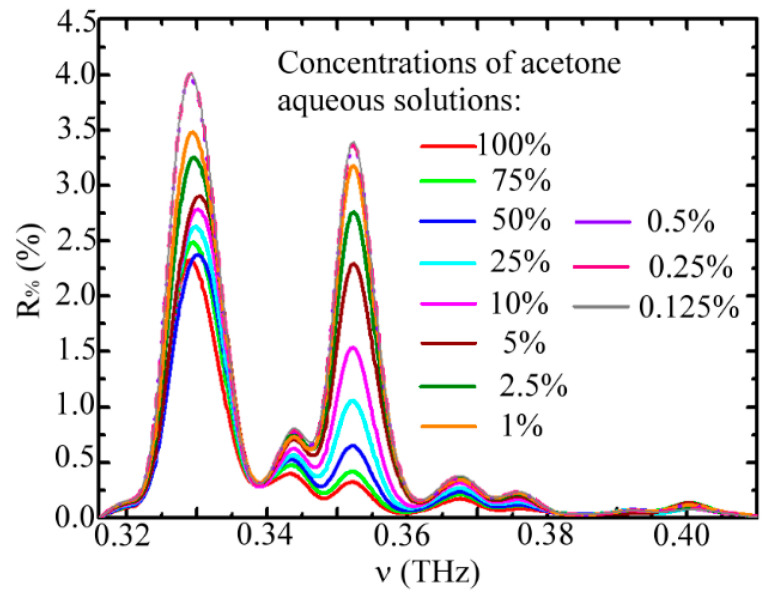
Reflectivity spectra of the WGS unit for various concentrations of acetone aqueous solutions.

In the experiment, the waveguide modes at 0.329, 0.344, 0.352, and 0.368 THz frequencies, shown as the spectral peaks in Figure 2, were used for sensing vapor samples inside the pipe core and comparing the sensing abilities among these four frequencies. The spectral main peaks at 0.329 THz and 0.352 THz frequencies were the core modes (Equation (1)), and those at 0.344 THz and 0.368 THz frequencies were the cladding modes (Equation (2)). Their gas-sensing abilities were characterized by the spectral reflectivity (*R*_%_) of the WGS unit under different densities of acetone vapor exposure, which is illustrated in Figure 3. The acetone vapor densities or molecular amounts in the unit volume of the pipe core space were approximately proportional to the acetone liquid concentrations. Thus, *R*_%_ values decreased with increasing acetone concentrations of aqueous solutions. Figure 3 illustrates that the *R*_%_ decrement of core modes (0.329 THz and 0.352 THz frequencies) was more evident than that of cladding modes (0.344 THz and 0.368 THz frequencies). Two factors influence the spectral response of WGS reflectivity. One is the frequency-dependent overlap volume between the waveguide modal field and vapor sample inside the pipe core. The other is the frequency-dependent molecular absorption for THz radiation. For the WGS scheme, the pipe field–vapor overlapping volume is the major factor leading to such distinct *R*_%_ values between the core and cladding modes, which is found from further analysis as follows.

**Figure 4:**
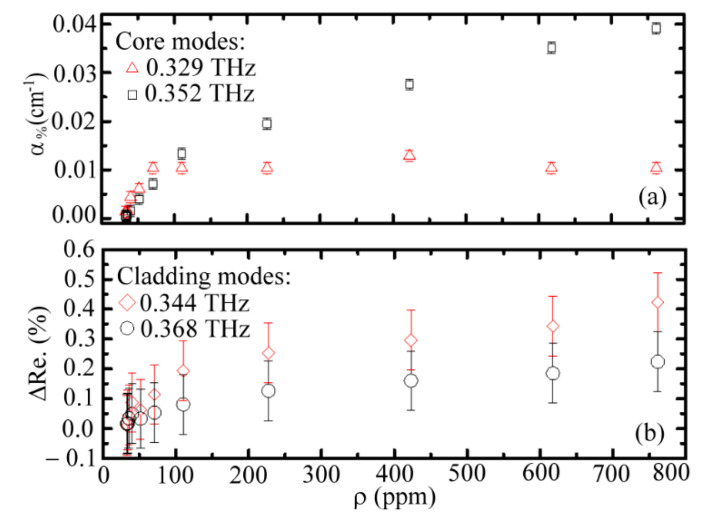
(**a**) Loss coefficients of acetone vapor at core mode frequencies, and (**b**) reflectivity variation of cladding mode frequencies under different concentrations of acetone vapor exposure.

According to Equations (9)–(12), the loss coefficients of a WGS unit, *α*_%_, at the core mode frequencies are dominated by the molecular absorption of the acetone vapor. The criteria are the relatively low losses of pipe guidance, metal reflection, and air absorption inside the pipe core. Figure 4a shows the estimated absorption coefficients of core modes (*α*_%_) for different vapor molecular densities (*ρ* values) inside the WGS unit (Equation (12)). The molecular absorption of acetone is approximately proportional to *ρ*, but the absorption apparently saturates for a *ρ* value above 110 ppm. The saturation effect at 0.329 THz was more evident than that at 0.352 THz. However, the highest/saturated *α*_%_ value of the 0.329 THz wave was approximately 0.01 cm^−1^, which occurred at a low *ρ* value of 70 ppm. In contrast, the *α*_%_ value of the 0.352 THz wave still slowly increased while the vapor density was greater than 110 ppm. For the 0.352 THz wave, the highest *α*_%_ value occurred at the *ρ* value of 771 ppm and was equal to 0.039 cm^−1^.

The radial power distributions of the 0.329 THz and 0.352 THz waves were calculated by the finite-difference time-domain (FDTD) method. The fractional powers inside the core for the 0.329 THz and 0.352 THz waves were estimated at approximately 94% and 96%, respectively, based on the FDTD calculation. The power ratio in the pipe core of the 0.352 THz wave was higher than that of the 0.329 THz wave. The core fractional power difference of 2% is critical to enhance the frequency-dependent *α*_%_ through a long waveguide propagation length (i.e., 60 cm). Thus, the 0.352 THz wave has a high attenuation response (Figure 4a, *α*_%_) for sensing the acetone vapor inside the pipe core.

The cladding modes have strong guidance loss in the WGS unit, and their *α*_%_ values of cladding mode frequencies are not reliable in Equation (12). Thus, the reflectance variation (Δ*Re.*) was used instead of *α*_%_ to characterize the frequency-dependent absorption of acetone vapor. Figure 4b expresses that the high-frequency cladding mode at 0.368 THz had a relatively lower Δ*Re.* than that of the low-frequency wave at 0.344 THz for each available vapor density. This result also arises from the difference of fractional power inside the core to interact with the acetone vapor. However, the Δ*Re.* values of both cladding-mode frequencies encountered difficulty in identifying all of the *ρ* values, that is, the Δ*Re.* values are smaller than the error bars as *ρ* changes. The low visibility of Δ*Re.* to identify different molecular densities (*ρ*) for the cladding modes mainly originates from their extremely lower power fraction inside the core than those of the core modes (Figure 4a). The FDTD calculation showed that the average fractional power inside the core of these cladding modes (0.344 THz and 0.368 THz waves) was approximately 58%, which was lower than that of core modes (94% or 96%). Therefore, the cladding modes are unsuitable as sensing waves.

### 3.3. Sensing Ability for Vapors of Various Volatile Liquids

**Figure 5:**
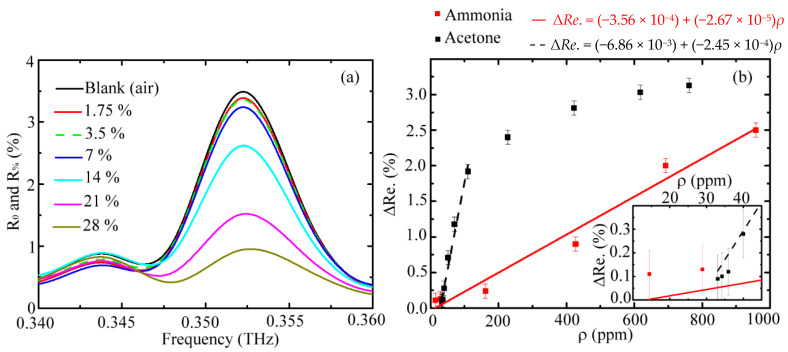
(**a**) Reflectivity spectra of a WGS unit for blank condition (*R*_0_) and loading various concentrations of ammonia aqueous solutions (*R*_%_). (**b**) Δ*Re.* response at 0.352 THz for acetone and ammonia vapors with different *ρ* values. (Insertion) Δ*Re.* response of 0.352 THz for *ρ* values less than 45 ppm of acetone and ammonia vapors.

For acetone vapor sensing, the 11th-order cladding and core fields had higher sensitivities at the 0.344 THz and 0.352 THz frequencies, respectively (Figure 4). To study the THz response of the waveguide-based sensing scheme for different types of vapors, we loaded another vapor, ammonia, into the WGS unit (Figure 1). Different *ρ* values were prepared from the dilution of a 28% ammonia aqueous solution, and the corresponding reflectance spectra of the WGS unit were illustrated in Figure 5a. All of the *R*_%_ values were lower than the *R*_0_ value and almost linearly decreased as vapor concentration increased at a spectral frequency of 0.352 THz because the THz wave power dissipation resulted from the absorption of ammonia vapor. In contrast, the *R*_%_ values at the cladding mode frequency, 0.344 THz, were random at various liquid concentrations of ammonia (Figure 5a), which was unlike the proportional absorption response of acetone vapor in Figure 4. This result indicates that the 11th-order core mode, 0.352 THz, is more applicable for identifying various vapors, which was compared with the cladding mode of 0.344 THz.

Figure 5b summarizes the Δ*Re.* responses of acetone and ammonia vapors for different *ρ* values in the WGS unit. The Δ*Re.* values of ammonia molecules proportionally increased with the *ρ* value within 0–959 ppm. For the acetone vapor sensing, the linearly proportional response of Δ*Re.* was only within the low vapor density region of 0–110 ppm, which is consistent with the response of *α*_%_ value in Figure 4a. These proportional responses are illustrated by linear fitting curves in Figure 5b. The slopes of the fitting curves represent the detection sensitivity of the waveguide mode of a glass pipe, which is equal to the Δ*Re.* response per 1 ppm of gas molecules. From the slopes of the linear fitting curves and error bars of Δ*Re.* (Figure 5b), the detection limits of *ρ* value variation were estimated as approximately 4 and 37 ppm, respectively, for the acetone and ammonia vapor detection within the linear response concentration range. The error bars were evaluated from the analysis of power stability at 0.352 THz, monitored by the WTR unit within one minute and corresponding to 0.1% deviation in average (Δ*Re.*). In the experiment, the minimum detectable vapor density was measured at 36 ppm for acetone vapor sensing. As shown in the insertion in Figure 5b, the Δ*Re.* values for different vapor *ρ* values lower than 36 ppm were not identified from the system noise. This means that the sensitivity of 4 ppm, namely the detection limit of the vapor density change, can only be performed in the acetone vapor density range of 36–110 ppm. For ammonia, the detection limit of 37 ppm sensitivity was performed only for the vapor density above 160 ppm.

According to the analysis of the Δ*Re.* response, the acetone molecules had a higher sensitivity than the ammonia molecules at approximately one order. The sample information in Table 1 further showed that the vapor density of ammonia was higher than that of acetone. However, the molecular dipole moment of acetone is approximately double that of ammonia. Therefore, the power dissipation of the 0.352 THz wave, contributed by the molecular polarity factor (i.e., molecular dipole moment, *p*, in Equation (8)), is larger than that by the density factor *ρ* (Equation (8)).

**Figure 6:**
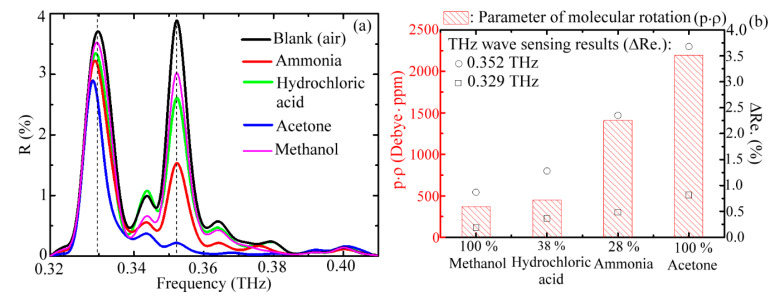
(**a**) Reflectivity spectra of a WGS unit for blank condition and loading various vapors, and (**b**) THz response in terms of Δ*Re.* at the 0.329 THz and 0.352 THz frequencies for different vapors with various molecular rotation abilities represented by *p* × *ρ*.

In addition to the dipole moment, the parameters related to the rotational inertia (i.e., molecular weight) and molecular quantity of vapor molecules should both be considered to identify various types of vapors. As the THz radiation would externally perturb a whole vapor molecule to form field–dipole interaction, it changes the original molecular rotation or vibration state [14,15]. A parameter that is defined by the product term, *p* × *ρ*, with a unit of Debye ∙ ppm, was applied to study the molecular rotation in response to the THz wave absorption. Figure 6a illustrates the measured *R*_%_ spectra of various vapors including methanol, hydrochloric acid, ammonia, and acetone molecules, whose molecular specifications are listed in Table 1. The *R*_%_ values of core modes at 0.352 THz and 0.329 THz were monitored in the sensing experiment due to the largest overlap volumes between the modal field and vapor samples, which are denoted by the dashed lines in Figure 6a. Comparison of the *R*_%_ and *R*_0_ values at the two core mode frequencies showed that the corresponding Δ*Re.* values were used to identify the vapor samples. Figure 6b illustrates the sensing results of Δ*Re.* for the 0.352 THz and 0.329 THz core modes, which are denoted by hollow circles and square dots, respectively. The sequence of Δ*Re.* values at 0.352 THz and 0.329 THz was acetone > ammonia > hydrochloric acid > methanol. This finding is consistent with the sequence of the rotational parameter (*p* × *ρ*) of the vapor molecule represented by the histogram in Figure 6b. The reflectivity spectrum of the blank condition (*R*_0_) in Figure 6a is slightly different from that of Figure 2 for the absolute peak and relative values at the two core-mode frequencies. The same situation occurred in the 100%-volume concentration of acetone vapor as shown in the comparison represented by the blue curve of Figure 6a and red curve of Figure 3. The difference was caused by the different *S* values (Figure 1c) while rearranging the WGS setup between the two individual experiments. This does not affect the sensing performance that recognizes the different types and concentrations of vapors because the mechanical parameter of *S* is fixed at the respective measurements. Therefore, the Δ*Re.* or *α*_%_ values can be related to the molecular properties for these two experiments.

**Figure 7:**
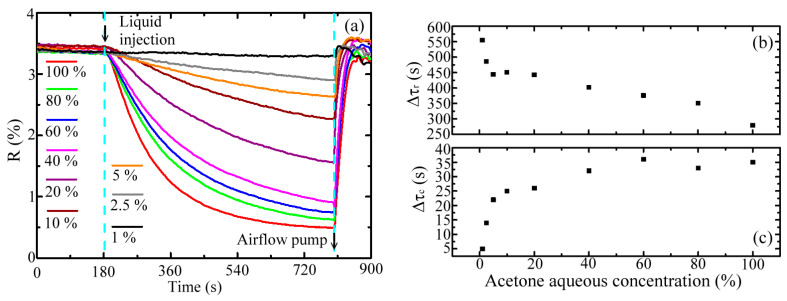
(**a**) Dynamic reflectivity of a WGS unit at 0.352 THz for various acetone aqueous solutions, and (**b**) response and (**c**) recovery times of the WGS unit.

Figure 7 shows the measured response and recovery times of the WGS unit for detecting various concentrations of acetone vapors at 0.352 THz, which evaporate from the 0.3 cm^3^-volume solutions with different acetone volume concentrations (1–100%). As shown in Figure 7a, the volatile liquid was injected at 180 s and the evaporated vapor was exhausted at 800 s by an air pump, as indicated by the cyan dashed lines in Figure 7a. Before 180 s, the WGS reflectivity was approximately constant around 3.5% under air exposure (i.e., blank pipe condition). The WGS reflectivity gradually decreased after sample injection. This arose from the gradual power dissipation of the pipe-guided THz radiation through the absorption of acetone vapor. Finally, the WGS reflectivity achieved a steady value while the complete interaction between THz radiation and acetone vapor occurred. The high concentration of vapor exposure has a steep and deep decrease in reflectivity because of the fast vapor diffusion. The response–recovery time for each vapor concentration is defined as the duration between 90% and 10% of the maximum reflectance decrease before–after 800 s (Figure 7a). Equation (13) defines the response time denoted as $\Delta \tau_{r}$ , wherein $\tau_{r-90\%}$ and $\tau_{r-10\%}$ indicate the times, respectively, for 90% and 10% of the maximum reflectivity variations during the power attenuation process (180−800 s).

(13) $$\Delta \tau_{r}=\tau_{r-90\%}-\tau_{r-10\%}$$

Equation (14) defines the recovery time, denoted as $\Delta \tau_{c}$ , wherein $\tau_{c-90\%}$ and $\tau_{c-10\%}$ indicate the times, individually, for 90% and 10% of the maximum reflectivity variations during the power increase process (800−900 s).

(14) $$\Delta \tau_{c}=\tau_{c-10\%}-\tau_{c-90\%}$$

Figure 7b illustrates that the response time apparently reduces when the vapor concentration increases, which is mainly determined by the diffusion speed of a gas analyte. The shortest response time approached five minutes for sensing 100% acetone. Figure 7c summarizes the recovery time for sensing various concentrations of acetone vapors. The recovery time was mainly decided by the air-flow rate of an air pump (23 L/min, Figure 1) and increased with the vapor concentration. Above 40% concentration of the aqueous acetone, the recovery time approximated a constant value, 30–35 s.

## 4. Conclusions

In summary, a gas-sensing modality and analysis method was experimentally demonstrated at the sub-millimeter wave regime. A dielectric pipe based on the antiresonant reflecting waveguide principle was used to recognize various categories and densities of vapors in a reflective manner. Within the available frequency range of the Gunn oscillator module, 0.316–0.408 THz, the most sensitive waveguide frequency for vapor detection was located at the high-order core mode frequency of 0.352 THz due to the largest field-analyte overlapped volume in the pipe core. Two kinds of vapors, acetone and ammonia, were prepared to externally diffuse into the pipe and accurately distinguished based on the Δ*Re.* response of the pipe-guided THz radiation. The low-ppm detection limit was realized experimentally for acetone vapor sensing. In the experiment, the minimum detectable vapor amount of acetone was 36 ppm and the detection limit of vapor density change was estimated to be 4 ppm for the wide response range of 36–110 ppm. Additionally, the THz response to four types of vapors was characterized experimentally and found to be approximately proportional to the molecular rotation parameter and molecular quantity. The simple reflection-type gas sensor based on a pipe offers sufficiently long interaction and high modal confinement for sensitive detection. No adsorber, molecular labeling, and extra gas cell exists for collection and increase of the gas concentration. Label-free gas sensing can easily be accomplished through the pipe-guided molecular probe, THz radiation. The demonstrated gas-sensing modality is potentially applicable to industrial gas-leak tracing, explosive detection, environmental pollutant monitoring, and medically exhausted breath detection.

## Figures and Tables

**Table 1:** Molecular properties of gas samples [29,30].

Sample	Contents			Vapor Pressure (kpa)	Vapor Density (*ρ*) (mg/L, ppm)	Molecular Dipole Moment (*p*) (debye)
	Volume Concentration	Molecule	Molecular Weight (g)			
Methanol	100%	CH_3_OH	32.0	16.8	216.75	1.70
Hydrochloric acid	38%	HCl	36.5	28.3	414.95	1.08
Ammonia	28%	NH_4_OH	35.0	67.97	957.97	1.47
Acetone	100%	CH_3_COCH_3_	58.0	32.53	761.54	2.88
